# Bio Pharmaceutics Classification System (BCS) Class IV Drug Nanoparticles: Quantum Leap to Improve Their Therapeutic Index

**DOI:** 10.15171/apb.2018.070

**Published:** 2018-11-29

**Authors:** Sachin Kumar, Ramneek Kaur, Rashi Rajput, Manisha Singh

**Affiliations:** Department of Biotechnology, Jaypee Institute of Information Technology Noida 201307, India.

**Keywords:** Hydrochlorothiazide, Bioavailability, Particle size analysis, Encapsulation efficiency, Permeability kinetics

## Abstract

***Purpose:*** Biopharmaceutics classification system (BCS) class IV compounds, exhibits least oral bioavailability, low solubility and intestinal permeability among all pharmaceutical classes of drugs. Thus, these drugs need more compatible and efficient delivery system. Since, their solubility in various medium, remains a limitation so, polymeric nano coacervates based drug loading with modified approach for them may prove to be a solution ahead. Therefore, in present study Chitosan is opted for encapsulating the BCS class IV drug (Hydrochlorothiazide) to attain better stability, enhanced permeability and lower toxicity.

***Methods:*** For this study, Hydrochlorothiazide (HCTZ) was opted for formulating chitosan based nano-coacervate system.

***Results:*** Optimized HCTZ nanocoacervates exhibited the average particle size of 91.39 ± 0.75 nm with Poly-dispersity index score of 0.159 ± 0.01, indicating homogeneity of colloidal solution. Zeta potential and encapsulation efficiency of HCTZ nanocoacervates were recorded as -18.9 ± 0.8 mV and 76.69 ± 0.82 % respectively. Further, from TEM and SEM evaluation the average particle size for the same were found in conformity (35-50 nm), with almost spherical morphology. Also, the EDX (Electron Dispersive X-ray) spectrometry and FT – IR analysis of optimized formulation indicated the balanced chemical composition and interaction between the polymeric molecules. The HCTZ nano coacervates showed the linear diffusion profile through the dialysis membrane.

***Conclusion:*** We can conclude from the present study that the optimized HCTZ nano coacervates may prove to be a suitable potential option for effective delivery of BCS class IV drugs.

## Introduction

The drug absorption rate in gastrointestinal (GI) tract is impacted by plenty of factors, like physicochemical nature, size and molecular weight of the compounds, metabolic, physiological functions, structure and surface of the gut cells etc.^1,2^ Notwithstanding this complexity, the Bio pharmaceutics Classification System (BCS) developed by Amidon et al.^3^ and Lipinski et al., prominently indicated that the synthetically derived drug leads, enormously fabricated by introduction of high-throughput screening (HTS) and combinatorial chemistry but, on the other side they were facing challenges from poorly water soluble drugs.^4,5^ Based on the Bio pharmaceutics Classification System, drugs are classified into four categories depending on their solubility and permeability properties like class I compounds are the ones having higher solubility and permeability; class II representing lower solubility but higher permeability; class III showing higher solubility but less permeability; and lastly class IV compounds with very less count of solubility and permeability index.^3^ Afterwards when this classification system was deeply dwelled and studied, it came in to the light that drug formulation and their carrier system areequally responsible in determining the rate and extent of absorption in GIT, increasing the bioavailability and therapeutic index of the classified drugs. Now, several approaches for improving drug delivery, solubility and permeability are constantly designed and modified, specifically for class II and IV compounds. The approaches such as complexation, micronization, crystal modification, increasing the drug dissolution rate, higher solubilization of the drugs etc., are more explored but these techniques do have restrictions to improve the assimilation and permeability of class IV drugs. Consequently, the best solution to improve the bioavailability of these drugs would be to return to the lead optimization phase of drug discovery and alter their structures to obtain the appropriate physicochemical properties.^5,6^ Nevertheless, discovering a novel therapeutic agent is a tough, time consuming and high cost bearing approach. In addition, very less count of therapeutic compounds, out of the millions, being tested each day, reaches the market. Therefore, a more viable and effective approach to improvise and redesign the drug formulation with respect to its carrier system, encapsulation and its targeted release is focused upon. In the present study we have selected one such BCS class IV drug, Hydrochlorothiazide (HCTZ)^7^ which is a well categorized Thiazide diuretic, considered as the first-line of treatment for hypertension and listed as an essential medicine in WHO list.^8^ Although, it’s an essential medication but due to its low bioavailability (65%) low permeability^9^ and extensive hepatic metabolism, it loses its therapeutic efficacy. Hence, to evade all these limitations an up graded delivery mechanisms are required which can be attained by designing the polymer based nanoparticle drug delivery system, which is known to warrant safe and efficient delivery of active compounds and enhanced bioavailability. Consequently, drug delivery through nanocoacervates has widely emerged successfully in recent years and many ongoing researches have reported the enhanced pharmacodynamic and pharmacokinetic profiling of a drug. Hence, in the present study HCTZ coated nano coacervates system was designed by using chitosan, a linear polyamine, having free amine groups, readily available for cross linkage, anticipated to enhance the therapeutic index of HCTZ. The process of nano coacervation formulation depends upon the degree of hydration in the colloidal system, solubility of drug compound in various solubilizing mediums (aqueous, alkali, alkaline etc.) and then finally deposition of polymer coacervates around the surface of drug molecules.^9^

## Materials and Methods

HCTZ was obtained from Jubilant Life Sciences, India. Chitosan and Dialysis membrane (9777, M.W. 12,400 Da) was procured from Himedia Laboratories, Mumbai, India and Sigma Aldrich, USA. Acetic acid, NaOH and all other chemical used were of analytical grade.

### Preparation of HCTZ nano coacervates 

**Figure 1:**
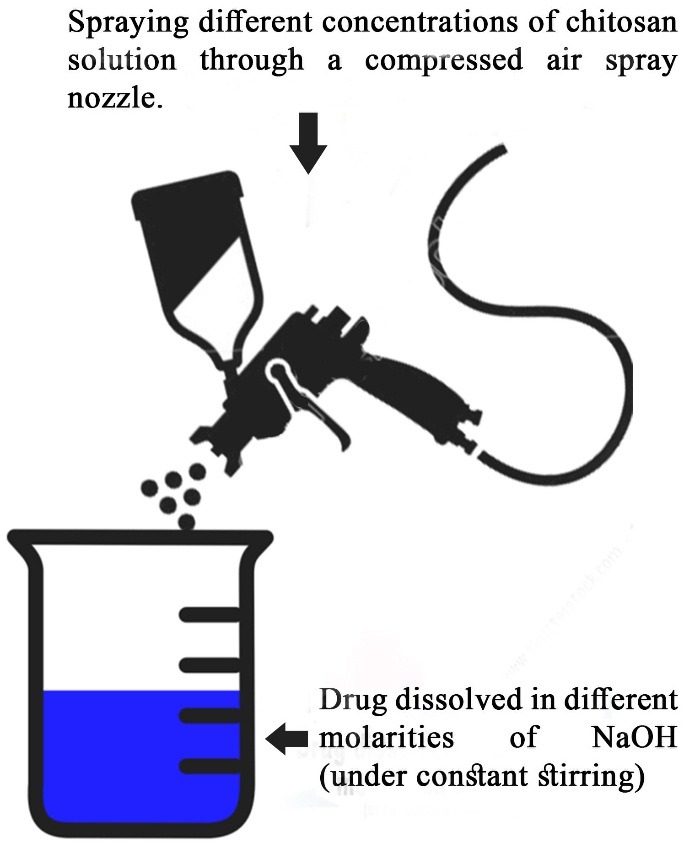
Image representing the chitosan nanocoacervates preparation by coacervation method.

Chitosan solution (1 - 2.5 mg/ml) was dissolved in 5% (v/v) glacial acetic acid and stirred overnight continuously at 2800Xg.Thereafter, HCTZ (6 mg/ml) was added in to NaOH solution of different molar concentrations (1M, 1.5M, 2M, 2.5M) as represented in Table 1, then through a high pressure compressed air spray (Pneumatic air spray nozzle), it was sprayed in chitosan solution, under continuous stirring, forming coacervates droplets in nanometric size range (Figure 1). Lastly, separation and purification of particles was done by centrifugation, followed by successive washing of coacervates solution with hot and cold water thrice.

The entrapment efficiency (EE) of HCTZ in the formulated coacervates system was determined by estimating the free drug available in the supernatant, after sonication (10 minutes) and centrifugation of the colloidal solution (40 minute,12750g speed). The supernatant was analyzed at 273 nm and the entrapment efficiency (EE) was calculated using the following equation:^10^

$$Encapsulation\;efficiency\;(\%)=\frac{CS_{D}-CSS_{D}}{CS_{D}}\times 100$$

Where, CS_D_ = Total loaded drug in chitosan solution and CSS_D_ = drug in supernatant

**Table 1:** List of ratio combination for HCTZ nano coacervates with varying degree of chitosan concentration and NaOH molarity

**Chitosan concentration (mg/ml)**	**Molarity of NaOH (M)**
1.0 – A	1.0 – A1
	1.5 – A2
	2.0 – A3
	2.5 – A4
1.5 – B	1.0– B1
	1.5 – B2
	2.0 – B3
	2.5 – B4
2.0 – C	1.0 – C1
	1.5 – C2
	2.0 – C3
	2.5 – C4
2.5 – D	1.0 – D1
	1.5 – D2
	2.0 – D3
	2.5 – D4

### Statistical optimization of formulated nanocoacervates

The statistical optimization of various process parameters for the experiment were done using Design-Expert® software (two-level full factorial experimental design).^11,12^ This experiment predicted the significance of interdependent parameters and estimated the listed effects of 4 experimental data- chitosan concentration, NaOH Molarity, NaOH: Chitosan ratio and sonication time on entrapment efficiency. The analysis was done by calculating variance to determine the significance of factors.^13^

### Characterization of optimized formulation

#### Particle size (PSA) and Zeta potential (ZPA) analysis of optimized nanocoacervates

The Zetasizer particle size measurement (Malvern Zetasizer 1000 HS, UK) was performed using Dynamic Light Scattering (DLS) method (also known as PCS - Photon Correlation Spectroscopy) which measures Brownian motion and relates this to the size of the particles by illuminating the particles with a laser and analyzing the intensity fluctuations in the scattered light.^14,15^ Then the sonicated and diluted (1:100) samples of drug loaded nanocoacervates (HCTZ NC’s) were subjected for particle size and zeta potential analysis.

#### Transmission Electron Microscopy (TEM)

TEM analysis was done to confirm the size range and morphological features of HCTZ NC’s. The interaction between electron beam and subjected test sample, forms an image which is further focused and magnified onto an imaging device called photographic film and then finally detected by a sensor. The optimized formulation (A1) was diluted 50 times and sonicated for 15 minutes. Then a drop of this sample was then fixed on 300 mesh carbon-coated copper grid with 2% of phosphotungstic acid (PTA) and analyzed at SAIF (Sophisticated Analytical Instrumentation Facility), Panjab University, Chandigarh, Punjab. The images of representative areas were taken at suitable magnifications (10,000x).

#### Scanning Electron Microscopy (SEM) and energy-dispersive spectroscopy (EDX)

The morphological and elemental analysis of optimized nano coacervates (A1) were corroborated by scanning electron microscopy (SEM) (ZEISS EVO 40) along with energy dispersive spectroscopy (EDX) (PANanalytical epsilon 5) scan respectively. The sample preparation was done by adding a drop of optimized HCTZ NP (A1) suspension on a metallic surface coated with gold layer, followed by, air drying under vacuum system and placing it under scanning grid for further analysis^16^ at TEM facility Amity University Noida.

#### Fourier Transform Infrared Spectroscopy (FTIR)

Fourier Transform Infrared Spectroscopy (IR-810, JASCO, Tokyo) was done to identify the functional groups present and interaction between the core molecules of HCTZ and outer polymeric shell. For analysis, the test samples were prepared by potassium bromide disc method^17^ and FT-IR spectra of HCTZ, optimized nanocoacervates without HCTZ and with HCTZ were scanned from 400-4000 cm^-1^ band width.

#### In vitro release kinetic studies

The *in vitro* release kinetics analysis was done to compare the pattern of drug (HCTZ) release through the dialysis membrane (Sigma 9777) in Franz diffusion cell. The activated dialysis membrane was mounted between the donor and receiver compartment. The donor compartment was filled with the test samples (HCTZ and HCTZ NP’s) alternatively and receiver compartment with PBS buffer (pH 7.4) and kept on continuous stirring for 10 hours. The diffused samples were collected after every 30minutes of time interval from the outlet port of receiver compartment and were compensated with equal volume of fresh PBS to maintain the equilibrium state. Then absorbance of test samples was taken at 280nm.

## Result and Discussion

### Preparation and optimization of HCTZ coacervates

**Figure 2:**
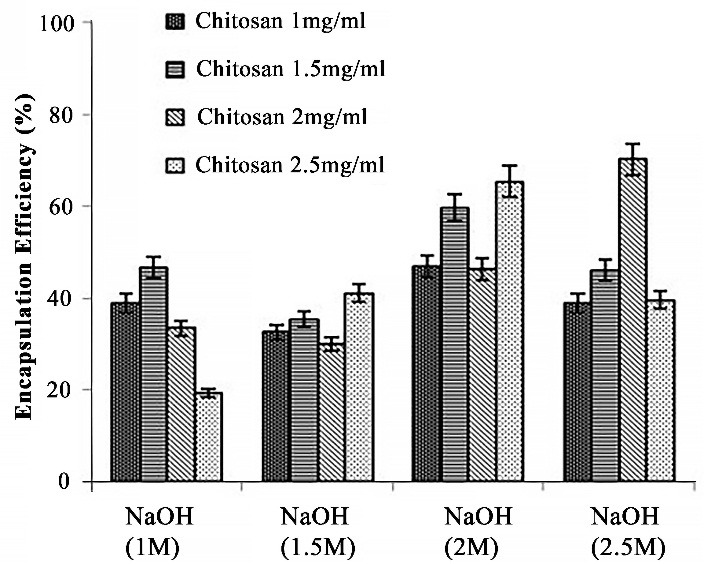
Comparative analysis of different chitosan concentrations and NaOH molarity based on entrapment efficiency.

After ratio optimization of chitosan volume and concentration, different formulations of nanocoacervates were prepared. The comparison between different NaOH molarity in each optimized chitosan concentration (based on entrapment efficiency) was done (Figure2). It was been observed that drug entrapment incremented with increasing chitosan concentration and NaOH Molarity but decreased after certain range (Chitosan concentration - 2mg/ml, NaOH - 2.5M), reflecting the possibility of increased resistance created by higher degree of NaOH molarity, hence preventing the chitosan from drug encapsulation. Stable nanocoacervates were obtained in formulation C4 having the chitosan and NaOH ratio of 2:2.5 (C2, NaOH 2.5) with highest entrapment efficiency of 76.69± 0.82%.^18,19^

### Statistical analysis

The process parameters involved in formulating the HCTZ NP’s were evaluated statistically by using two-level factorial design (Stat-Ease Design Expert version 10) to identify the evident significance of optimized parameters with respect to the entrapment efficiency of HCTZ loaded nanocoacervates. It has been observed that the maximum entrapment efficiency of 76.69% (11^th^ run*) and minimum of 46.07% (4^th^ run*) was recorded from the designed model system which was in accordance with the experimental data (Table 2). Moreover, ANOVA analysis results confirmed the significance of model system (p value 0.0008< 0.05) with 99% of confidence interval for mean (Table 3). However, the f value for the same was calculated to be 30.07, predicting the noise probability of 0.08%. It’s also been reported that higher proximity of R^2^ value towards 1, highlights the model strength; hence R^2^value for designed model system was recorded as 0.9836 confirming the higher interdependence of the model parameters.

*Run = statistically designed and calculated combination of entered process parameters for analysis

**Table 2:** Runs obtained from two-level factorial experiment.

**Run**	**A-Chitosan**	**B-NaOH Molarity**	**C-NaOH : Chitosan**	**D-Sonication Time**	**Entrapment Efficiency**
1	1.5	2	0.5	15	59.63
2	2	2	0.5	10	58.57
3	2	2	0.5	15	61.18
4	1.5	2.5	1	15	46.07
5	2	2.5	1	15	68.73
6	2	2.5	0.5	15	71.46
7	1.5	2	1	15	52.43
8	2	2	1	15	46.33
9	1.5	2.5	0.5	15	49.53
10	1.5	2	0.5	10	60.54
11	2	2.5	0.5	10	76.69
12	1.5	2	1	10	55.5
13	1.5	2.5	0.5	10	51.5
14	1.5	2.5	1	10	48.52
15	2	2.5	1	10	68.09
16	2	2	1	10	49.94

**Figure 3:**
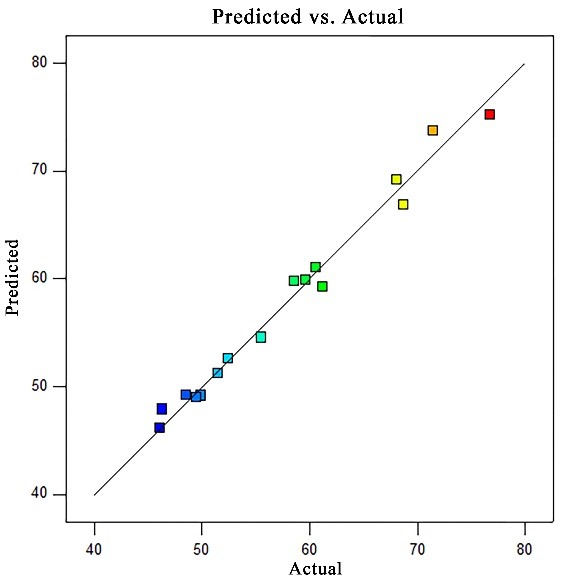
Predicted vs actual graph for entrapment efficiency of HCTZ loaded nanocoacervates

Also, the regression equation for the model system supports the higher dependability and relevance of process parameters with respect to response (entrapment efficiency).^20^ The first order polynomial equation in the form of factor codes is as follows:

Further, graphical representation of the estimated and recorded entrapment efficiency of HCTZ nanocoacervates (Figure 3) showed the correlation between all the optimized parameters and hence, confirms the successful preparation of HCTZ loaded nanocoacervates.^21^

Entrapment Efficiency=+57.79+4.83*A+2.28*B-3.34*C-0.87*D+6.34*AB-1.01*AC+0.18*AD+1.12

### Particle size and Zeta potential analysis of optimized nanocoacervates

**Figure 4:**
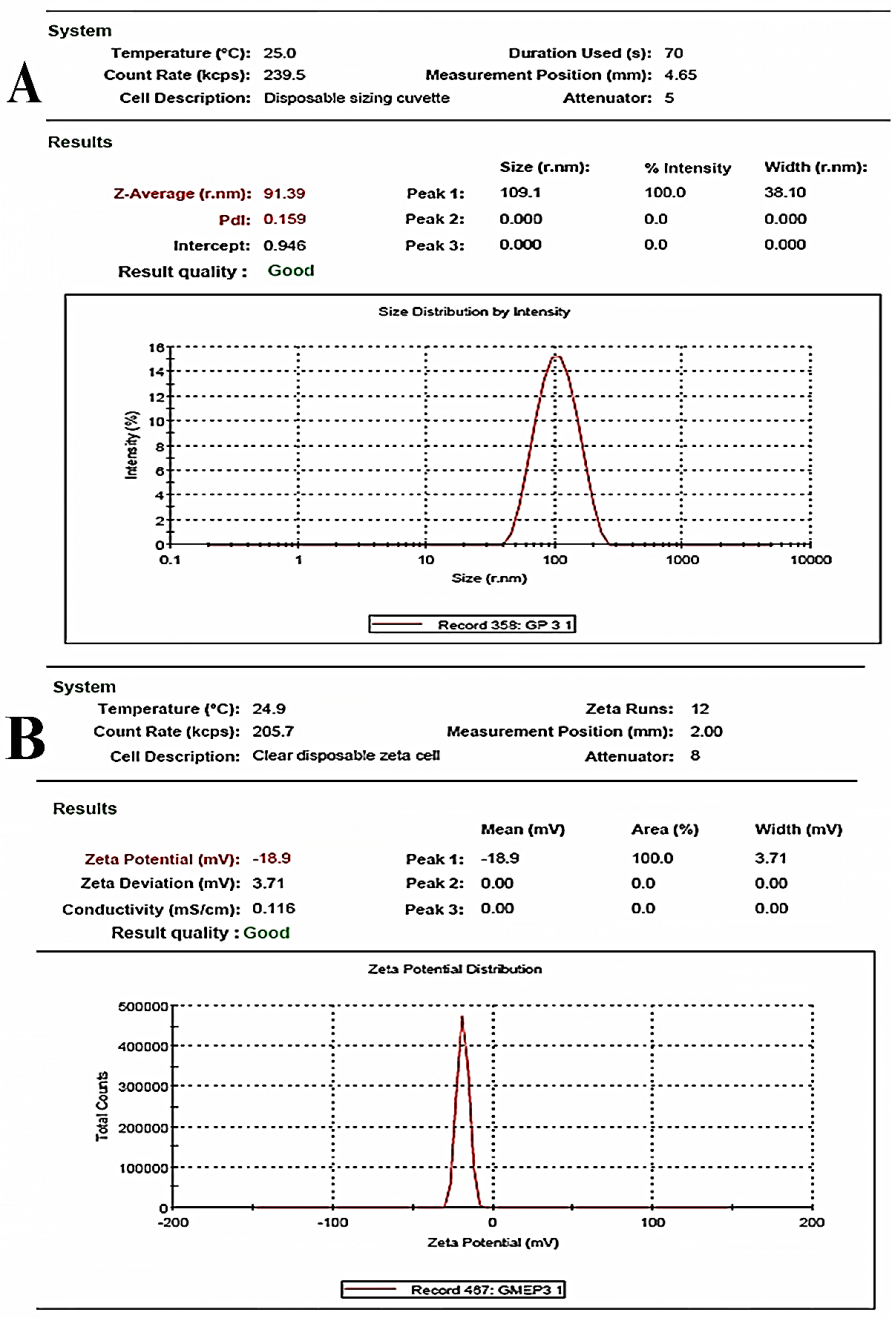
Graphs depicting the particle size (A) and zeta potential (B) of optimized formulation (C4) of HCTZ NPs (Hydrochlorothiazide nano-coacervates).

The average particle size (PSA) of the optimized nanocoacervates (C4) were recorded as 91.39 ± 0.34 nm, suggesting the possibility of easy penetration through the various biological barriers and poly dispersibility index (PDI) score of 0.159 ± 0.047 indicating the higher dispersibility and homogeneity of the coacervates in colloidal solution (Figure 4(A)). Moreover, the zeta potential (ZP) of the same was noted as -18.9 ± 0.8 mV representing the negative surface electrical charge due to some dissociated surface groups (carboxyl and/or amino groups) (Figure 4(B)). The nanocoacervates were showing higher stability with less molecular charge which falls under the range of ± 30 mV and enhance the non-aggregation properties of nanocoacervates as reported in earlier studies.^22^

### Transmission Electron Microscopy (TEM)

**Figure 5:**
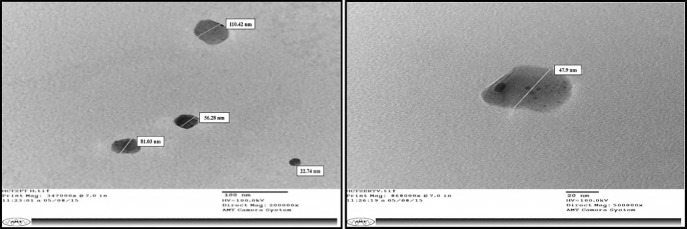
Transmission Electron Spectroscopy (TEM) micrographs showing particle size and morphology of optimized formulation (C4) at magnification scale of 200nm and 20nm.

The size of HCTZ encapsulated nanocoacervates were further scanned with transmission electron microscopy (TEM) at different magnification scales (100nm and 20nm) and they exhibited the size range between 35-50 nm (in diameters) (Figure 5) indicating that size of most of the nanocoacervates is below 100 nm, thus in capacitating the nanocoacervates to permeate through most of the biological barrier due to its minimum surface area.^23^

### Scanning Electron Microscopy (SEM)

**Figure 6:**
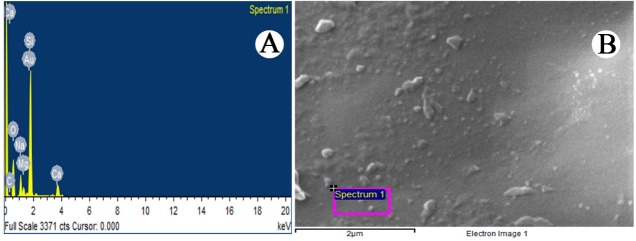
Image of scanning electron micrograph (SEM) of optimized nanocoacervates (HCTZ NPs) showing the surface network.

Scanning Electron Microscopy (SEM) was used for the morphological characterization of particles. SEM uses a high energy electron which is scanned over the surface and the back scattering of the electrons is analyzed thereafter.Samples were coated by spraying gold powder to enhance its conductivity. The results displayed almost spherical and smooth morphology of nanocoacervates at 200 nm (Figure 6).^24^

### Energy Dispersive X- Ray Spectrometry (EDX)

**Figure 7:**
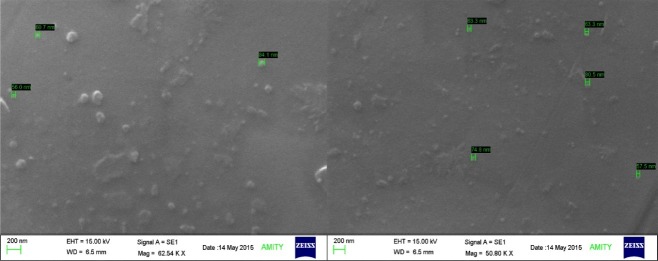
EDX analysis showing (A) localized chemical composition and (B) topological image of nanocoacervates.

In Electron Dispersive X-ray Spectrometry, a focused electron beam is bombarded on the solid sample which emits an X-ray spectrum of localized chemical.^25^The EDX scanning (Figure 7) exhibited various peaks indicating the presence of C, O and Au and the existence of carbon (C) suggests the formation of chitosan nanocoacervates along with these forms there were certain more peaks of molecules (silicon, calcium, sodium, magnesium) noticed, suggesting existence of some water based impurities.^26^

### FTIR Analysis

**Figure 8:**
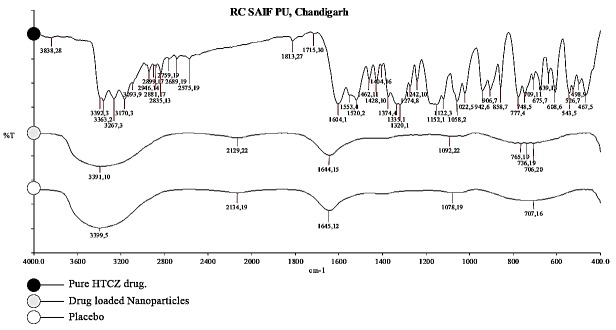
FT-IR spectra analysis for HCTZ drug, HCTZ NPs and only chitosan nanocoacervates.

The FT-IR spectra of HCTZ, optimized nanocoacervates without drug and optimized nanocoacervates with drug showed that the chemical structure of nanocoacervates is chitosan. The FTIR spectrum of plain HCTZ (Figure 8) illustrates peaks at 3362, 3267, and 3170 cm^-1^ assigned to NH and NH_2_ stretching. It also shows peaks at 1602 cm^-1^ and 1520 cm^-1^ corresponding to the heterocyclic ring system, and peaks at 2361 and 2339 cm^-1^ assigned to C-H stretching of the thiazide ring.^24,27^ In addition, it also showed a peak at 1321cm^-1^ corresponding to SO_2_ asymmetric stretching and at 1174 and 1152 cm^-1^ corresponding to SO_2_ symmetric stretching.^28^

### In vitro release kinetics

**Figure 9:**
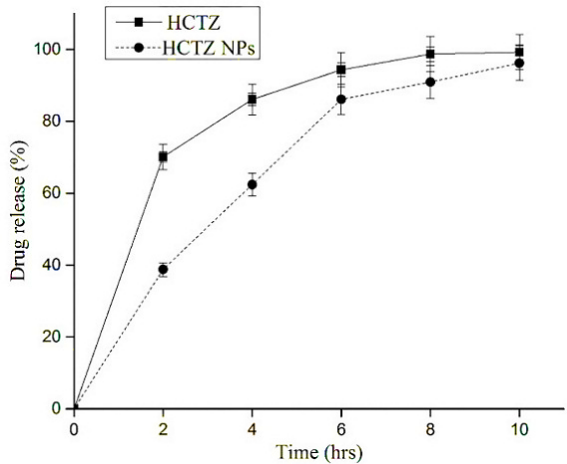
*In vitro* release kinetic analysis for HCTZ drug, HCTZ NCs and only chitosan nanocoacervates.

*In vitro* release kinetics of HCTZ and HCTZ NCs was studied to compare the permeability through the dialysis membrane. It was observed that 99.26 ± 0.14% release of HCTZ after 10 hours whereas, in the case of HCTZ NCs it was 96.19 ± 0.21% release in the same time limit (Figure 9), indicating a typical linear diffusion profile through the dialysis membrane. Also, graphical representation exhibited the burst release in case of HCTZ pure (70.12 ± 0.17%) till 2 hours which was reduced in HCTZ NC’s (38.79 ± 0.31%), and was observed to be more linear. However, the cumulative percentage of release for HCTZ NCs (90.92 ± 0.07%) was attained at 8 hours and after that it was sustained till 10 hours. However, for HCTZ the cumulative percentage of release (94.34 ± 0.09%) was attained at 6 hours and thus, leads to maximum release of drug content.

## Conclusion

The designed HCTZ loaded nanocoacervates carrier system (HCTZ NCs), showed efficient maximum encapsulation of (76.69 ± 0.82%) and the nanometric size (35 to 50 nm) with spherical morphology. Further characterization (TEM, SEM and EDX) analysis also confirmed its desired morphological surface structure and FTIR scans confirmed no significant surface interaction between polymer and drug.The *in vitro* permeability analysis exhibited sustained drug release pattern up to 10 hours, leading to enhanced therapeutic effects for longer duration. These coacervates comparatively became more stable and shielded the drug compounds from enzymatic degradation than the pure drug and being smaller in size (nanometric), is anticipated to easily permeate through biological barriers and act as a potential carrier system for targeted drug delivery, although needs to be validated on various systems. This nanocoacervates system, therefore, represents a significantly viable approach to achieve enhanced therapeutic efficiency at low dosage, but require more detailed pharmacological assessments for clinical applications.

## Acknowledgments

The research group is grateful to the Department of Biotechnology Jaypee Institute of Information Technology Noida (U.P.), SAIF (Sophisticated Analytical Instrumentation Facility), Panjab University, Chandigarh, Punjab, SMITA Research Lab Indian Institute of Technology, New Delhi and Department of Biotechnology Amity University Noida (U.P.) for providing necessary facilities to execute this work.

## Ethical Issues

Not applicable.

## Conflict of Interest

The authors declare no conflict of interest.
